# Multi-Omics Analysis Reveals Age-Dependent Metabolic Remodeling and Immune Maturation in the Cecum of Liangshan Yanying Chickens

**DOI:** 10.3390/vetsci13060594

**Published:** 2026-06-18

**Authors:** Zengwen Huang, Jing Wang, Chaoyun Yang, Heng Yang, Zhiqiang Hu, Gang Shu, Zengpeng Lv, Dayong Si

**Affiliations:** 1 College of Animal Science, Xichang University, Xichang 615000, China; xcdaxue@xcc.edu.cn (Z.H.);; 2 College of Tourism and Urban-Rural Planning, Xichang University, Xichang 615000, China; 3 College of Veterinary Medicine, Rongchang Campus, Southwest University, Chongqing 402460, China; 4 College of Veterinary Medicine, Sichuan Agricultural University, Chengdu 611130, China; 5 College of Animal Science and Technology, China Agricultural University, Beijing 100193, China

**Keywords:** Liangshan Yanying chicken, brooding period, cecum, metabolomics, molecular regulatory network

## Abstract

Liangshan Yanying chicken is a valuable plateau-adapted indigenous poultry breed in China. The poultry cecum modulates nutrient metabolism, gut microbial colonization and intestinal immune barrier establishment, while the molecular mechanisms driving its age-dependent development during the brooding stage remain unclear. Here, integrated transcriptomic and metabolomic profiling coupled with bioinformatics correlation analysis were conducted on cecal samples collected from chickens at post-hatching days 1, 14 and 28. Significant temporal changes were observed in cecal gene expression and metabolite abundance, and day 14 was identified as a critical window for cecal functional maturation and microbial colonization. In total, 2424 metabolites were annotated, including 600 differentially accumulated metabolites. The cecum exhibited phase-specific metabolic patterns: endogenous energy metabolism dominated at 1–14 d, while lipid biosynthesis prevailed at 14–28 d. The intestinal IgA immune network was verified as the core pathway maintaining cecal immune homeostasis in young chicks. Multi-omics conjoint analysis yielded 53 overlapping KEGG pathways, 14 core pathways, 3 pivotal metabolites and 5 hub genes, based on which three interactive regulatory networks were constructed. Transcriptomic data were validated via qRT-PCR. This study reveals cecal metabolic remodeling and regulatory characteristics during the brooding period, supplementing gut developmental research on plateau indigenous chickens. Notably, these results reflect age-related cecal developmental changes rather than breed-specific high-altitude adaptation mechanisms. Further independent verification is required for metabolomic data and predicted regulatory networks. This finding provides a theoretical basis for scientific breeding and feeding management of Liangshan Yanying chickens.

## Simple Summary

Liangshan Yanying chicken is a precious plateau-adapted indigenous chicken breed in China. The cecum governs gut metabolism, microbial colonization and intestinal immunity, whereas its age-dependent developmental mechanisms during brooding remain unclear. This study performed combined transcriptomic and metabolomic analysis on cecal samples from chickens at post-hatching days 1, 14 and 28. Day 14 was confirmed as the critical period for cecal maturation. The cecum showed phase-specific metabolic features, and the IgA immune network sustained intestinal immune homeostasis. Multi-omics analysis identified key regulatory molecules and pathways. These cecal variations are age-related developmental changes instead of high-altitude adaptation traits. This work provides theoretical support for the scientific feeding and breeding of this plateau chicken breed.

## 1. Introduction

Liangshan Yanying chicken, a rare local poultry breed in China, was obtained the protection of Agricultural Product Geographical Indication in 2010 and was included in the List of Animal Genetic Resources in China, thus possessing important germplasm conservation value and unique research value [1]. As a dual-purpose breed for meat and egg production, this chicken inhabits the closed high and semi-high mountain areas of Liangshan Prefecture and has been artificially selected by the Yi ancestors for generations. Such long-term artificial selection has endowed the breed with superior meat quality, strong disease resistance, and adaptability to alpine, humid, hypoxic environments, as well as roughage feeding conditions. This indigenous dual-purpose breed exhibits good production performance and strong stress tolerance under plateau rearing conditions. The present study explores the molecular mechanisms underlying cecal development of Liangshan Yanying chickens during the brooding period. The results can also support the development of local characteristic animal husbandry, which is consistent with the diverse value attributes of indigenous livestock and poultry germplasm resources in China [2].

The intestinal tract serves as the core organ for poultry to resist extreme environments and maintain organismal homeostasis, and the cecum, as a key component of the intestinal tract, performs multiple functions, including crude fiber fermentation, secondary nutrient absorption, microbial colonization and immune barrier construction. The developmental status of the cecum directly affects the growth performance and environmental adaptability of poultry [3,4]. Existing studies have confirmed that poultry can regulate somatic energy metabolism and immune response through metabolic products of cecal microorganisms to adapt to complex living environments [5,6]. For instance, lactic acid bacteria in the cecum of Tibetan chickens can regulate the intestinal microecology to enhance high-altitude adaptability [7], and the specific expression of the inducible nitric oxide synthase gene provides an important reference for the study of hypoxic adaptation mechanisms in poultry [8]. However, the molecular regulatory network of the cecal functional system during the brooding period in Liangshan Yanying chickens remains unclear, especially the linkage mechanism between genes and metabolic phenotypes. Notably, this study only explores age-related cecal changes under a single altitude condition, and no comparative tests with lowland breeds or different altitude groups were conducted.

Transcriptomics can accurately reveal the dynamic changes in gene expression, while metabolomics can directly reflect the characteristics of somatic metabolic phenotypes. Integrated analysis of the two omics can connect the regulatory link between *genes* and *metabolic phenotypes*, thereby providing technical support for elucidating complex biological mechanisms. At present, multi-omics technologies have been widely applied in the research field of the poultry intestinal tract. Studies such as the analysis of high-altitude adaptation mechanisms of cecal microorganisms in Tibetan chickens, the revelation of high-altitude adaptation rules of Tibetan chickens through multi-omics analysis of the gut-organ axis [9], and the clarification of the correlations among host gene expression [10], intestinal microbiota and metabolites in chickens by integrated multi-omics analysis have all verified the reliability and effectiveness of these technologies [11], and thus provided a mature technical paradigm for this study.

In this study, cecal tissues of Liangshan Yanying chickens at different developmental stages were used as experimental materials, and an integrated analysis of transcriptomics and metabolomics was conducted to systematically screen differentially expressed genes and differential metabolites, construct the molecular regulatory network and core pathways of cecal development, and elucidate the molecular mechanisms of cecal tissue development in Liangshan Yanying chickens. The results fill the research gap in the cecal development mechanisms of plateau poultry, provide a theoretical basis for the breed protection and genetic improvement of Liangshan Yanying chickens, and also offer a reference for the research on environmental adaptation mechanisms of other plateau livestock and poultry.

## 2. Materials and Methods

### 2.1. Ethical Approval

All animal experiments were implemented in strict compliance with the Guidelines for the Care and Use of Laboratory Animals formulated by the Animal Care and Use Committee of Xichang University, China. The experimental protocol was approved by the Animal Care and Use Committee of Xichang University (Approval No.: xcc2022003). Every effort was made to minimize animal suffering and reduce the number of experimental animals used, in strict adherence to the 3R principles (Replacement, Reduction, Refinement).

### 2.2. Experimental Animals and Sample Collection

A total of 200 fertilized eggs of Liangshan Yanying chickens were obtained from Yuexi County Xinyi Ecological Breeding and Planting Professional Cooperative, Liangshan Prefecture, Sichuan Province, China. All eggs were disinfected, weighed and individually numbered, then incubated synchronously in a Fuhui integrated incubator-hatcher (Wuhan Fuhui Co., Ltd., Wuhan, China). The incubation parameters were set as follows: a temperature of 37.8 °C and a relative humidity (RH) of 60% with egg turning every 2 h from day 1 to day 18 of incubation; the temperature and RH were adjusted to 37.2 °C and 70%, respectively, during the hatching period (day 19 to day 21). Egg candling was carried out on day 7, day 11 and day 19 of incubation to remove infertile eggs, early embryonic death eggs and late embryonic death eggs, resulting in a hatching rate of 82.5% (165 viable chicks hatched from 200 fertilized eggs). After hatching, 120 healthy chicks were selected for rearing with four replicate cages and 30 chicks per cage, and extra chicks were reserved as backups. Chicks were raised in standardized 2 m × 1 m cages under a cage-rearing system, with each cage regarded as an independent experimental unit. Ad libitum access to feed and water was provided for chicks following a staged feeding program, with a pre-starter diet fed from day 1 to day 14 and a starter diet from day 15 to day 28. Both diets were provided by Kunming Bangyun Feed Co., Ltd., Kunming, China, and complied with the Chinese Feeding Standard for Chickens (NY/T 33-2004), with detailed nutritional compositions presented in Supplementary Table S1. Daily cleaning of litter and feces and cage disinfection were performed to maintain good hygienic conditions, and no routine immunization was administered to ensure consistent experimental conditions for all chicks.

At 1, 14 and 28 days of age, 10 healthy chicks were randomly selected from each age group, respectively. Chicks were euthanized by isoflurane inhalation anesthesia followed by jugular vein exsanguination. Under sterile conditions, cecal tissue samples were immediately collected into sterile cryopreservation tubes, snap-frozen in liquid nitrogen, and subsequently stored at −80 °C for subsequent omics analysis.

For metabolomic analysis, 10 biological replicates per age group were adopted to reduce the deviation caused by individual metabolic differences. For transcriptome sequencing, considering the high sequencing depth, stable technical repeatability of RNA-seq platform, and the strict quality control of RNA samples, we set 3 biological replicates per group, which is the conventional sample size for poultry intestinal transcriptome research. Meanwhile, we evaluated the Pearson correlation between transcriptome samples (r ≥ 0.85) and PCA clustering results, which proved good sample homogeneity. Although a small number of replicates may slightly reduce statistical power for screening weakly differentially expressed genes, the main differentially expressed genes with significant expression changes can be reliably identified in this study.

### 2.3. Metabolite Extraction and Screening

Cecal tissue samples were freeze-dried using a Scientz-100F vacuum freeze dryer (Scientz Biotechnology Co., Ltd., Ningbo, China). The freeze-dried samples were pulverized for 1.5 min at a frequency of 30 Hz with zirconia beads in a Retsch mixer mill (Model MM 400, Retsch GmbH, Haan, Germany). Precisely 50 mg of freeze-dried powder was weighed and dissolved in 1.2 mL of 70% (*v*/*v*) methanol solution. The mixture was vortexed for 30 s, and this vortexing step was repeated six times at 30-min intervals. After centrifugation at 12,000 rpm for 3 min, the supernatant was filtered through a 0.22 μm pore-size filter membrane (Cat. No. SCAA-104; ANPEL Laboratory Technologies, Shanghai, China).

The filtrate was subjected to metabolite analysis using ultra-performance liquid chromatography-tandem mass spectrometry (UPLC-MS/MS). The analytical system consisted of an ExionLC™ AD UPLC system and an Applied Biosystems 6500 QTRAP mass spectrometer (both from SCIEX, Framingham, MA, USA). Metabolite data processing was performed using Analyst 1.6.3 software (AB SCIEX, Framingham, MA, USA). Principal component analysis (PCA) and orthogonal partial least squares-discriminant analysis (OPLS-DA) were employed to identify differential metabolites among different age groups. Metabolites with variable importance in the projection (VIP) ≥ 1.0, fold change (FC) ≥ 2 or ≤0.5, and *p* < 0.05 were identified as significantly differential metabolites.

### 2.4. RNA Sequencing and Functional Annotation

Transcriptome sequencing of cecal tissue samples included four key steps: RNA extraction, RNA quality assessment, library construction and high-throughput sequencing, which were performed on cecal tissue samples collected at three developmental stages (1, 14 and 28 days of age). The integrity of total RNA extracted from cecal tissues was evaluated using 1% agarose gel electrophoresis and a NanoDrop spectrophotometer (Thermo Fisher Scientific, Wilmington, DE, USA).

Sequencing libraries were constructed using the NEBNext^®^ Ultra™ RNA Library Prep Kit for Illumina (New England Biolabs, Ipswich, MA, USA) in accordance with the manufacturer’s standard protocols. Library sequencing was conducted by MetWare Biotechnology Co., Ltd. (Wuhan, China) on the Illumina high-throughput sequencing platform, generating 150 bp paired-end reads. For functional annotation of unigenes, unigene sequences were aligned against the NR, KEGG, GO, Swiss-Prot, COG/KOG and TrEMBL databases using DIAMOND v2.1.25 and BLASTX(NCBI BLAST+v2.17.0)software [12]. In addition, the amino acid sequences predicted from unigenes were annotated against the Pfam database using HMMER software (Hammer 3) to obtain conserved domain information of the encoded proteins.

### 2.5. Transcriptome Data Analysis

Raw sequencing reads were preprocessed using fastp software [13] to ensure high data quality. Paired-end reads were filtered out according to the following criteria: reads containing N bases accounting for more than 10% of the total length; reads containing more than 50% low-quality bases (Q-value ≤ 20). For samples with biological replicates, differential expression analysis between groups was performed using DESeq2 software [14,15] to generate differential gene expression profiles. After differential expression analysis, the Benjamini-Hochberg method was used to adjust the *p*-values for multiple hypothesis testing, thereby estimating the false discovery rate (FDR). Genes with |log_2_ (Fold Change)| ≥ 1 and false discovery rate (FDR) < 0.05 were defined as significantly differentially expressed genes (DEGs).

### 2.6. Weighted Correlation Network Analysis and Gene Network Visualization

Weighted gene co-expression network analysis (WGCNA) was performed on the gene expression profiles [16] of cecal tissues from Liangshan Yanying chickens at the three developmental stages. Pearson correlation coefficient (PCC) analysis was conducted between the identified co-expression modules and 18 cecal tissue metabolites to screen modules with the strongest correlations with metabolite changes. Genes with high correlation coefficients were selected to construct a gene co-expression network, which was subsequently visualized using Cytoscape 3.8.2 software (Cytoscape Consortium, San Diego, CA, USA).

### 2.7. Real-Time Quantitative PCR (qRT-PCR)

Genes involved in cecal tissue biosynthesis and regulatory genes were selected from RNA-seq data for expression validation using qRT-PCR, with chicken β-actin as the internal reference gene. Gene-specific primers were designed using Primer 5.0 software based on the existing transcriptome assembly results. qRT-PCR experiments were performed on a real-time fluorescence quantitative PCR instrument (Analytik Jena AG, Jena, Germany) with THUNDERBIRD Next SYBR qPCR Mix as the fluorescent dye. The qPCR reaction system (20 μL) contained 10 μL SYBR GREEN qPCR Master Mix, 0.4 μL forward primer (10 μmol/L), 0.4 μL reverse primer (10 μmol/L), 7.2 μL RNase-free water and 2 μL cDNA template. Relative gene expression was quantified via the 2^−ΔΔCt^ method. Primer sequences for all validated genes are provided in Supplementary Table S2. Each qRT-PCR reaction was run in three technical replicates. Raw data sorting was performed in Microsoft Excel 2021. Statistical analysis and figure generation were conducted with GraphPad Prism 9.5.1, and multi-omics data processing was completed using TBtools v1.137.

### 2.8. Screening of Core Pathways, Key Metabolites and Hub Genes

A self-established multi-dimensional weighted scoring system was used to screen 14 core KEGG pathways, 3 pivotal metabolites and 5 hub genes. Five evaluation dimensions were set with fixed weights, and the total score ranged from 0 to 10 points. Evaluation dimensions and weights: ① Inter-group differential significance (30%): pathways/metabolites/genes met the screening thresholds of *p* < 0.05, FDR < 0.05 and VIP ≥ 1.0; ② Temporal expression continuity (25%): molecules with sustained differential expression in all three comparison groups (D1 vs. D14, D14 vs. D28, D1 vs. D28); ③ Multi-omics correlation (20%): Pearson correlation coefficient (PCC) ≥ 0.7 and *p* < 0.05 between genes and metabolites; ④ Functional relevance (15%): molecules/pathways closely related to cecal development, intestinal metabolism, immune function and mucosal barrier; ⑤ Literature support (10%): molecules/pathways reported in previous poultry intestinal studies. Scoring rule and threshold: Each dimension was scored 0–10 points according to compliance. The final total score = Σ (single dimension score × corresponding weight). Molecules/pathways with a total score ≥ 7.0 were included in the candidate set. Molecules with irregular fluctuating expression/abundance were eliminated manually. Screening procedure: Firstly, 53 shared KEGG pathways from multi-omics data were scored and ranked, and the top 14 pathways were defined as core pathways. Secondly, metabolites associated with the 14 core pathways were scored, and 3 key metabolites were screened out. Finally, combined with network topology (node degree), 5 hub genes were determined from differential genes linked to core pathways and metabolites.

### 2.9. Statistical Analysis

All experimental data were analyzed using SPSS 26.0 software (IBM Corp., Armonk, NY, USA), and experimental graphs were generated using Origin 9.0 software (OriginLab Corp., Northampton, MA, USA). Significant differences between groups were determined using one-way analysis of variance (ANOVA) followed by Duncan’s multiple range test. Statistical significance was set at *p* < 0.05, * *p* < 0.01 and ** *p* < 0.001.

## 3. Results

### 3.1. Metabolite Identification and Data Quality Control in Cecal Tissues of Liangshan Yanying Chickens

**Figure 1:**
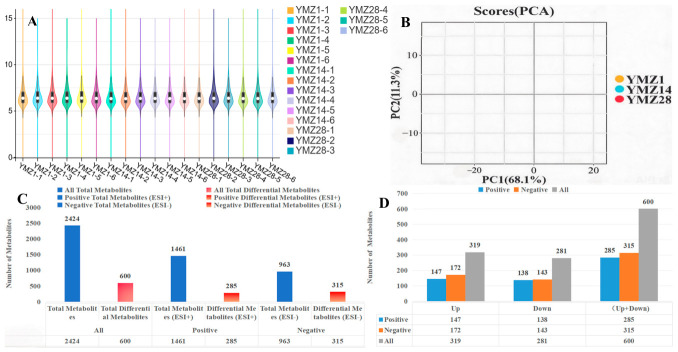
Metabolite identification and data quality control of cecal tissue in Liangshan Yanying chickens. (**A**) Metabolite identification results of cecal tissue; (**B**) Principal component analysis (PCA) of metabolites in cecal tissue of Liangshan Yanying chickens at different ages; (**C**) Statistical analysis of annotated metabolites; (**D**) Statistical analysis of differential metabolites in cecal tissue at different ages.

To elucidate the characteristics of metabolic remodeling in the cecum of Liangshan Yanying chickens during the brooding period, widely targeted metabolomic detection was performed on a total of 18 cecal tissue samples from chickens at 1, 14 and 28 days of age (D1, D14, D28). Data quality control results showed that the relative standard deviation (RSD) of metabolite abundance of all samples was ≤5% with good uniformity in density distribution, indicating high repeatability and stability of the detected metabolomic data (Figure 1A). Principal component analysis (PCA) results revealed that the contribution rates of principal component 1 (PC1) and principal component 2 (PC2) were 42.3% and 27.8%, respectively; samples at different ages were clearly clustered into three independent clusters with significant inter-group separation and tight intra-group aggregation, confirming that the metabolite composition of cecal tissues exhibited obvious temporal differences with the growth and development of brooding chickens (Figure 1B). A total of 2424 metabolites were identified in cecal tissues, among which 1461 (60.3%) were identified in positive ion mode and 963 (39.7%) in negative ion mode. The types of identified metabolites covered amino acids, lipids, carbohydrates, nucleotides and secondary metabolites, with the detection coverage significantly superior to that of similar poultry-related studies (Figure 1C). Further screening identified 600 differential metabolites among samples of the three age groups (285 in positive ion mode and 315 in negative ion mode), including 319 cumulatively upregulated metabolites and 281 cumulatively downregulated metabolites (Figure 1D), suggesting that the cecal tissues of Liangshan Yanying chickens were in a state of dynamic metabolic remodeling throughout the brooding period.

### 3.2. Screening and Stage-Specific Analysis of Differential Metabolites in Cecal Tissues at Different Ages

**Figure 2:**
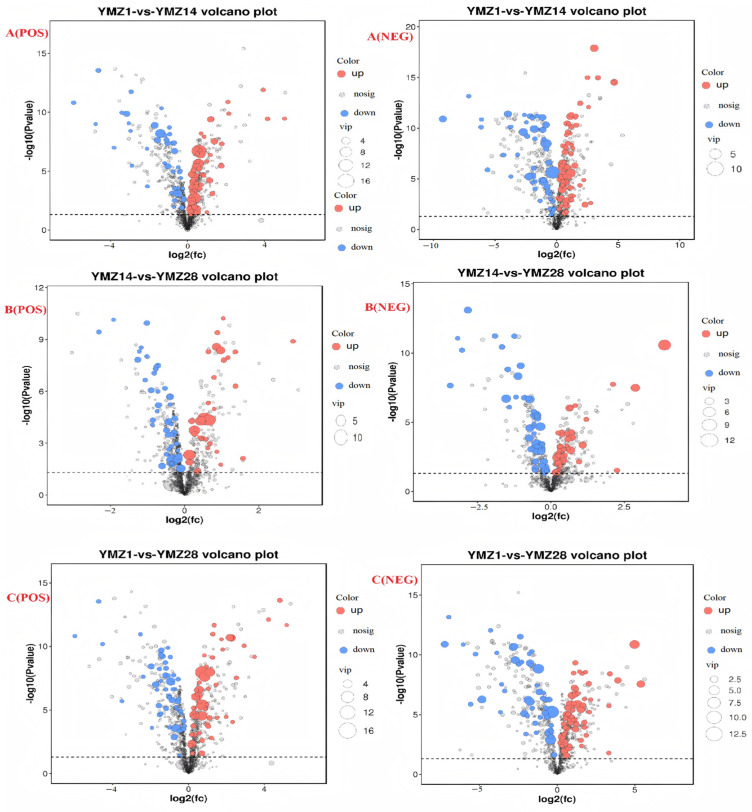
Volcano plot analysis of differential metabolites in cecal tissue of Liangshan Yanying chickens at different ages. (**A**) (POS/NEG) Volcano plot analysis of differential metabolites in cecal tissue of Liangshan Yanying chickens at 1 day vs. 14 days of age in positive/negative ion modes; (**B**) (POS/NEG) Volcano plot analysis of differential metabolites in cecal tissue of Liangshan Yanying chickens at 14 days vs. 28 days of age in positive/negative ion modes; (**C**) (POS/NEG) Volcano plot analysis of differential metabolites in cecal tissue of Liangshan Yanying chickens at 1 day vs. 28 days of age in positive/negative ion modes.

A dual screening criterion of orthogonal partial least squares-discriminant analysis variable importance in the projection (OPLS-DA VIP) ≥ 1 combined with Student’s *t*-test *p* < 0.05 was adopted for differential metabolite analysis of three sample groups (D1 vs. D14, D14 vs. D28, D1 vs. D28), so as to clarify the metabolic characteristics of cecal tissues at different stages during the brooding period. A total of 219 significantly differential metabolites were screened in the D1 vs. D14 group (127 upregulated and 92 downregulated) (Figure 2A), among which the significantly upregulated metabolites were mainly short-chain fatty acid derivatives and amino acids such as glutamic acid and aspartic acid, while the significantly downregulated metabolites were glycogen derivatives and bile acid precursors. This result indicates that the cecum gradually shifts its metabolic pattern from relying on endogenous energy reserves to utilizing exogenous nutrients, which is consistent with the physiological development of intestinal digestive and absorptive functions in newly hatched chicks. A total of 166 significantly differential metabolites were identified in the D14 vs. D28 group (75 upregulated and 91 downregulated) (Figure 2B); the downregulated metabolites were mainly intermediate products of amino acid metabolism, and the upregulated metabolites were dominated by lipid metabolism products such as unsaturated fatty acids and phospholipids, indicating that the metabolic focus of the cecum shifted to lipid synthesis and utilization at this stage, which was closely related to the maturation of the intestinal mucosal barrier and the increase in somatic energy storage. A total of 215 significantly differential metabolites were detected in the D1 vs. D28 group (117 upregulated and 98 downregulated) (Figure 2C), and these differential metabolites were concentrated in carbohydrate metabolism, amino acid metabolism and intestinal microbe-related metabolites, confirming that metabolic remodeling of the cecum runs through the entire brooding period with continuous adjustment of core metabolic pathways.

### 3.3. KEGG Functional Annotation and Core Pathway Enrichment Analysis of Differential Metabolites

**Figure 3:**
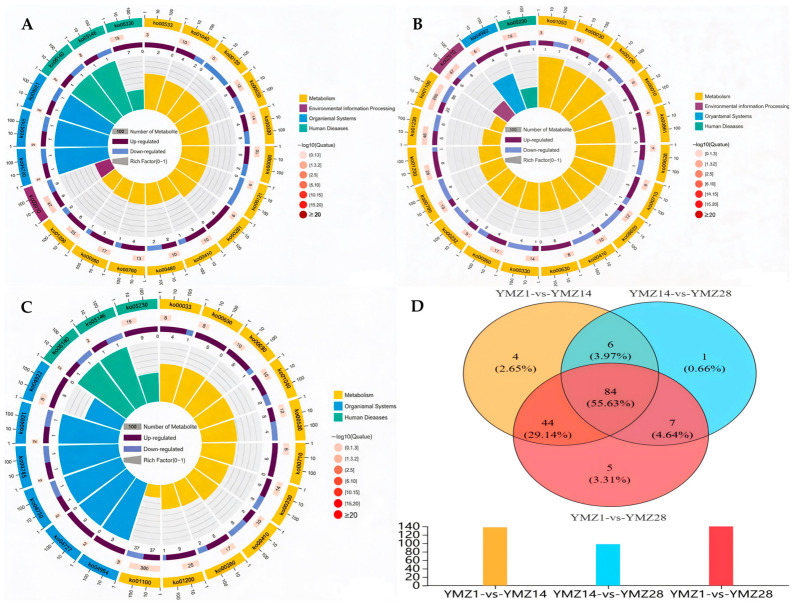
KEGG functional annotation and core pathway enrichment analysis of differential metabolites in cecal tissue of Liangshan Yanying chickens. (**A**) Core pathway enrichment analysis of differential metabolites in cecal tissue at 1 day vs. 14 days of age; (**B**) Core pathway enrichment analysis of differential metabolites in cecal tissue at 14 days vs. 28 days of age; (**C**) Core pathway enrichment analysis of differential metabolites in cecal tissue at 1 day vs. 28 days of age; (**D**) Integrated analysis of enriched pathways of differential metabolites in cecal tissue at different ages.

Based on the Kyoto Encyclopedia of Genes and Genomes (KEGG) database, functional annotation and pathway enrichment analysis (*p* < 0.05) were performed on differential metabolites to elucidate their biological functions and underlying regulatory mechanisms. Differential metabolites in the D1 vs. D14 group were enriched in 138 KEGG pathways, among which 32 were significantly enriched pathways; the core pathways included metabolic pathways (ko01100, enrichment rate 78.31%), microbial metabolism in diverse environments (ko01120, enrichment rate 22.89%) and biosynthesis of secondary metabolites (ko01110, enrichment rate 32.53%) (Figure 3A). The enrichment of microbial metabolism pathways suggests that the colonization process of intestinal microorganisms is officially initiated in chicks after hatching, and metabolic interactions between the microecology and the host begin to appear. Differential metabolites in the D14 vs. D28 group were enriched in 98 KEGG pathways, among which 27 were significantly enriched pathways with the same core pathways as the D1 vs. D14 group; the enrichment rate of the biosynthesis of secondary metabolites pathway increased to 37.84% (Figure 3B), suggesting that the secondary metabolic activity of the cecum was enhanced at this stage and participated in the improvement of intestinal mucosal immune function. Differential metabolites in the D1 vs. D28 group were enriched in 140 KEGG pathways, among which 35 were significantly enriched pathways with the enrichment rate of metabolic pathways reaching 79.57% (Figure 3C), further confirming that metabolic remodeling is the core regulatory mode for cecal tissues to adapt to the growth and development of brooding chickens. Venn diagram analysis showed that differential metabolites of the three groups were co-enriched in 84 KEGG pathways (Figure 3D), which were mainly basic metabolic pathways, amino acid metabolic pathways and microbe-related metabolic pathways, and thus served as the core pathways of cecal metabolic regulation during the brooding period.

**Figure 4:**
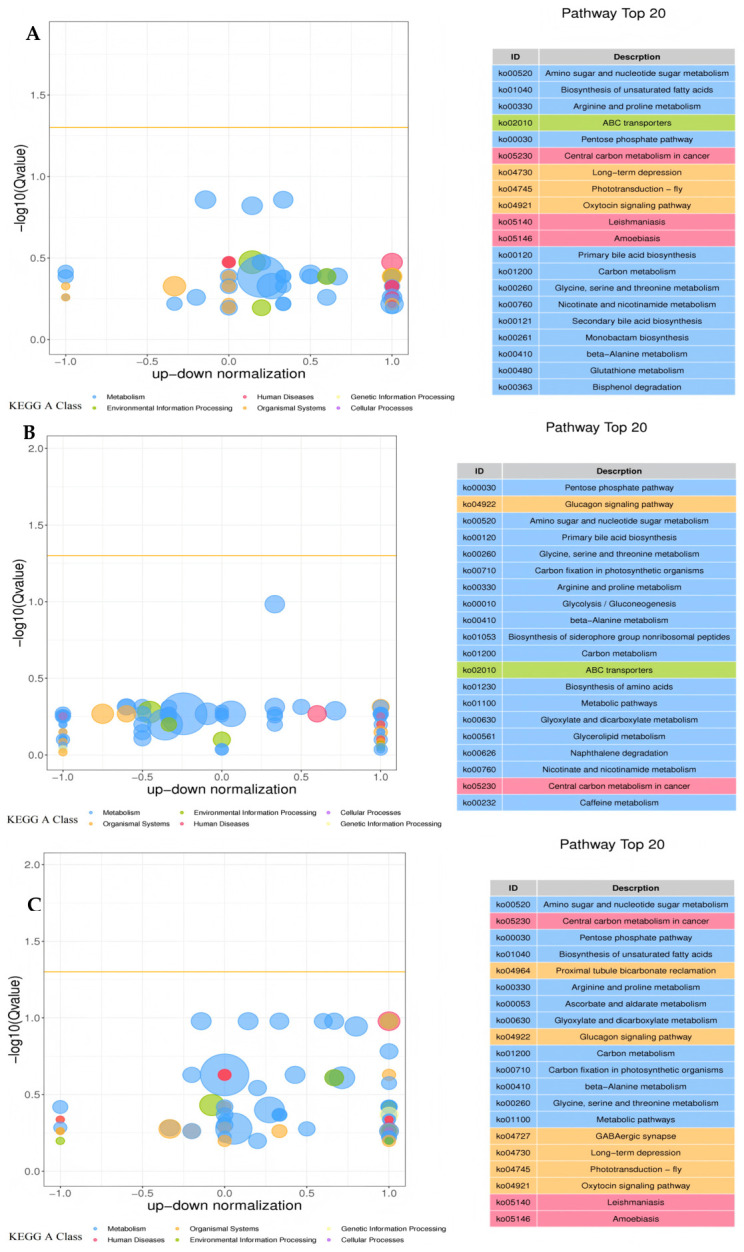
KEGG pathway significant enrichment analysis of differential metabolites in cecal tissue of Liangshan Yanying chickens. (**A**) Top 20 KEGG pathways significantly enriched with differential metabolites in cecal tissue at 1 day vs. 14 days of age; (**B**) Top 20 KEGG pathways significantly enriched with differential metabolites in cecal tissue at 14 days vs. 28 days of age; (**C**) Top 20 KEGG pathways significantly enriched with differential metabolites in cecal tissue at 1 day vs. 28 days of age.

Refined analysis of the top 20 significantly enriched pathways in each group found that amino sugar and nucleotide sugar metabolism (ko00520) was significantly enriched in all three groups, acting as a key pathway for cecal metabolic regulation during the brooding period; ABC transporters (ko02010) and other pathways were specifically significantly enriched in the D1 vs. D14 group, participating in the early establishment of the intestinal barrier; the pentose phosphate pathway (ko00030) was a significantly enriched pathway in the D14 vs. D28 group, which was closely related to the increased growth rate and elevated somatic energy demand of chicks (Figure 4A–C).

### 3.4. Mfuzz Clustering Analysis of Temporal Dynamic Trends of Differential Metabolites

**Figure 5:**
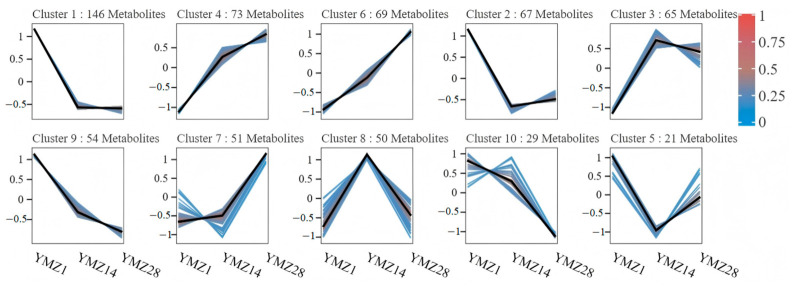
Mfuzz clustering analysis of temporal dynamic trends of differential metabolites in cecal tissue of Liangshan Yanying chickens.

Mfuzz fuzzy clustering analysis was used to elucidate the temporal expression patterns of differential metabolites from D1 to D14 to D28, which were divided into three core clusters. Cluster 1 contained 146 metabolites with a continuous decreasing trend from D1 to D28; these metabolites were mainly endogenous nutrients such as glycogen and amino acid derivatives, and the decrease in their abundance was associated with the enhanced nutrient absorption capacity of the intestinal tract of chicks and the reduced dependence on endogenous metabolites. Cluster 2 consisted of 73 metabolites showing an inflection-point increasing trend: their levels rose slowly from day 1 to day 14 and increased rapidly from day 14 to day 28. Most of these metabolites were microbe-related compounds, including short-chain fatty acids and secondary bile acids. These findings indirectly suggest that day 14 is a potential key node for the maturation of cecal microbial colonization. Since this conclusion is inferred from metabolomic data and no microbial sequencing was conducted in the present study, further verification is needed. Notably, metabolic interactions between the intestinal microecology and the host were markedly strengthened after this time point. Cluster 3 contained 69 metabolites with a continuous increasing trend from D1 to D28, which were primarily composed of lipid metabolism products such as unsaturated fatty acids and phospholipids that can provide structural support for the maturation of the cecal mucosal barrier and the synthesis of cell membranes (Figure 5).

### 3.5. Transcriptome Sequencing and Data Quality Control in Cecal Tissues of Liangshan Yanying Chickens

**Figure 6:**
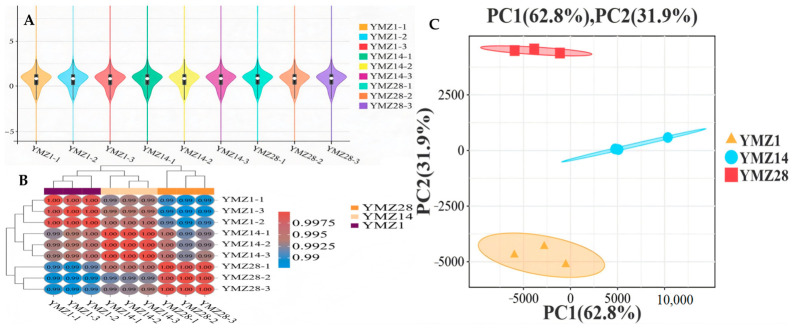
Transcriptome sequencing and data quality control of cecal tissue in Liangshan Yanying chickens. (**A**) Violin plot analysis of transcriptome sequencing data of cecal tissue; (**B**) Clustering analysis of transcriptome sequencing samples of cecal tissue; (**C**) Principal component analysis (PCA) of transcriptome sequencing data of cecal tissue at different ages.

To elucidate the molecular regulatory basis of cecal development, transcriptome sequencing (RNA-seq) was performed on cecal tissue samples at D1, D14 and D28 (three biological replicates per age group, 9 samples in total; see Section 2.2 for the explanation of sample size setting). Three biological replicates were used for transcriptome analysis; sample correlation and clustering results confirmed the reliability of the data, while limited replicates may have minor impacts on the detection of weak differential genes. Data quality control results showed that the Q30 value of all samples was ≥92%, the GC content was stable at 48~50% without obvious base preference, meeting the standards of high-quality sequencing; detailed data are presented in Supplementary Table S3. After standardization of gene expression levels by the FPKM method, the gene expression distribution trends of all samples were consistent (Figure 6A); the Pearson correlation coefficient between samples was ≥0.85 without obvious batch effects (Figure 6B); PCA results showed that samples at different ages exhibited tight intra-group aggregation and significant inter-group separation, with the most obvious separation between the D1 and D28 groups (Figure 6C). The above results confirm the high quality of the transcriptomic data in this study, which can be used for subsequent screening and functional analysis of differentially expressed genes.

### 3.6. Screening of Differentially Expressed Genes in Cecal Tissues at Different Ages

**Figure 7:**
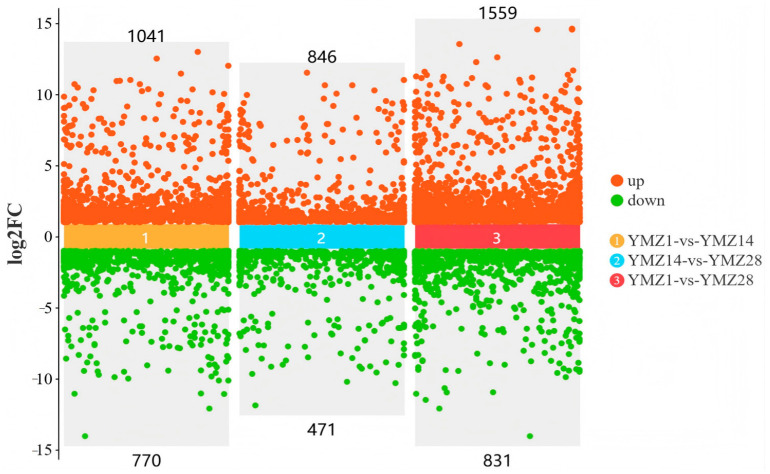
Statistical significance analysis of differentially expressed genes in cecal tissue of Liangshan Yanying chickens during brooding period.

**Figure 8:**
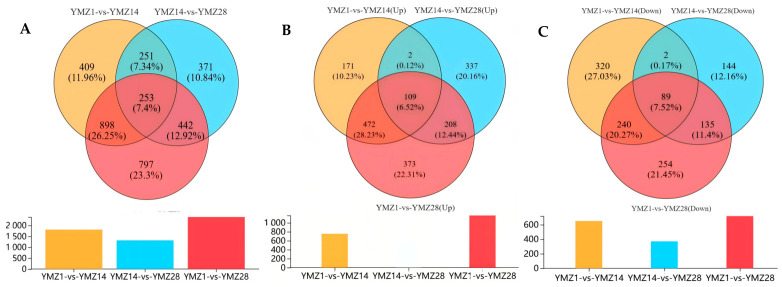
Screening of differentially expressed genes in cecal tissue of Liangshan Yanying chickens at different ages during brooding period. (**A**) Venn diagram analysis of all differentially expressed genes; (**B**) Screening results of upregulated differentially expressed genes in cecal tissue during the brooding period; (**C**) Screening results of downregulated differentially expressed genes in cecal tissue during the brooding period.

With |log2Fold Change (FC)| > 1 and false discovery rate (FDR) < 0.05 as the screening criteria, a total of 1811 differentially expressed genes (DEGs) were screened in the D1 vs. D14 group (1041 upregulated and 770 downregulated); 1317 DEGs in the D14 vs. D28 group (846 upregulated and 471 downregulated); and 2390 DEGs in the D1 vs. D28 group (1559 upregulated and 831 downregulated) (Figure 7). Venn diagram analysis found that 253 genes showed continuous differential expression in the three comparison groups, including 109 continuously upregulated genes and 89 continuously downregulated genes (Figure 8A–C). These genes serve as the core molecular targets regulating the early development of the cecum in Liangshan Yanying chickens, and their temporal expression characteristics directly drive the maturation of cecal morphology and function.

### 3.7. KEGG Enrichment Analysis of Differentially Expressed Genes

**Figure 9:**
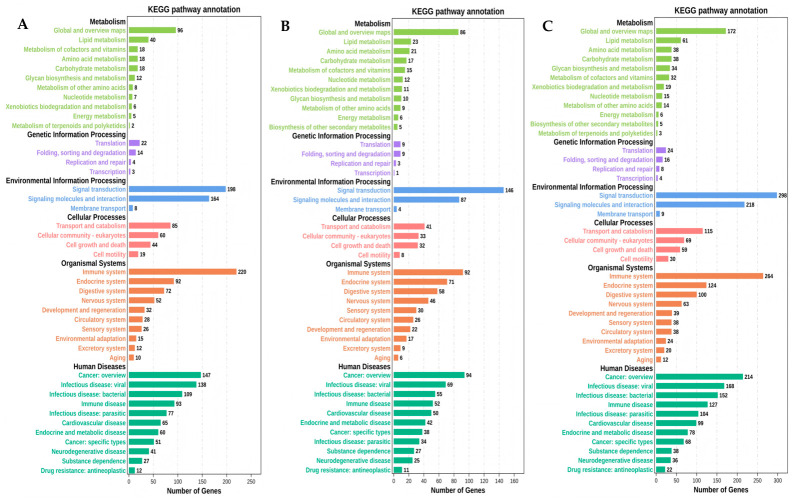
KEGG functional annotation of differentially expressed genes in cecal tissue of Liangshan Yanying chickens during brooding period. (**A**) KEGG pathway annotation of differentially expressed genes in cecal tissue at 1 day vs. 14 days of age; (**B**) KEGG pathway annotation of differentially expressed genes in cecal tissue at 14 days vs. 28 days of age; (**C**) KEGG pathway annotation of differentially expressed genes in cecal tissue at 1 day vs. 28 days of age.

**Figure 10:**
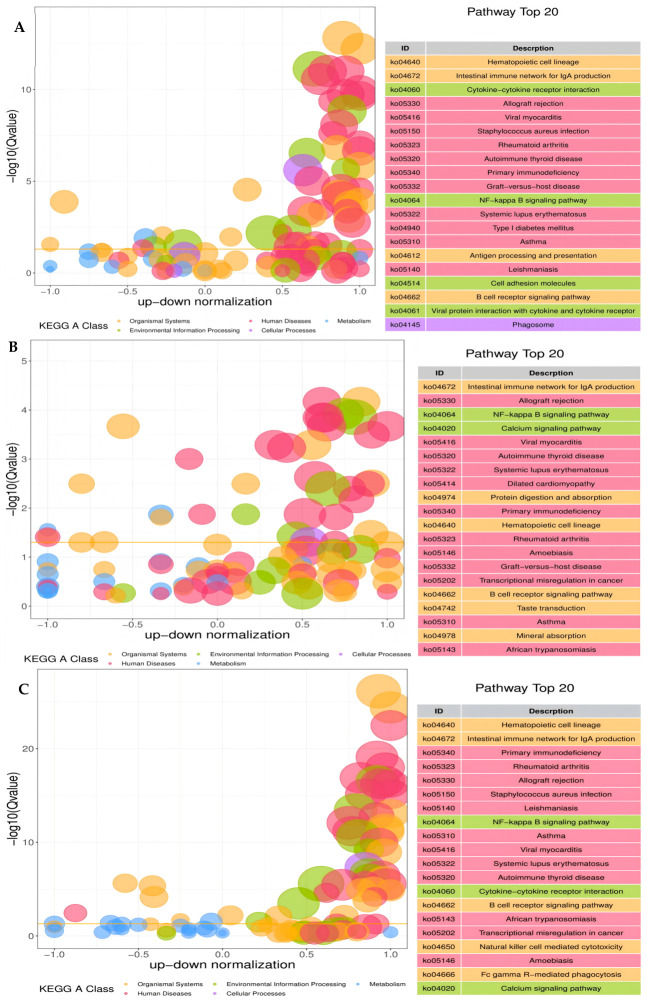
KEGG pathway significant enrichment analysis of differentially expressed genes in cecal tissue of Liangshan Yanying chickens. (**A**): Top 20 KEGG pathways significantly enriched with differentially expressed genes in cecal tissue at 1 day vs. 14 days of age; (**B**): Top 20 KEGG pathways significantly enriched with differentially expressed genes in cecal tissue at 14 days vs. 28 days of age; (**C**): Top 20 KEGG pathways significantly enriched with differentially expressed genes in cecal tissue at 1 day vs. 28 days of age.

KEGG annotation results showed that DEGs in the D1 vs. D14, D14 vs. D28 and D1 vs. D28 groups were annotated to 308, 314 and 330 KEGG pathways, respectively (Figure 9A–C), and 290 pathways were stably annotated throughout the brooding period, which are the core regulatory pathways of cecal development. Analysis of the top 20 significantly enriched pathways in each group revealed that the intestinal IgA immune network (ko04672) was markedly enriched across all three groups. This pathway acts as the core component for the establishment of cecal mucosal immunity during the entire brooding period. This pathway regulates the differentiation of intestinal B cells, synthesis and transport of secretory IgA, and further mediates the neutralization of harmful antigens and inhibition of pathogenic bacteria’s adhesion to epithelium. Hematopoietic cell lineage (ko04640) and cytokine-cytokine receptor interaction (ko04060) pathways were significantly enriched in the D1 vs. D14 group, indicating that 1–14 days of age is the key stage for the proliferation of intestinal immune cells and the initial construction of immune signal networks, which lays a foundation for the maturation of cecal immune function. (Figure 10A);Nuclear factor-κB (NF-κB) signaling pathway (ko04064) and calcium signaling pathway (ko04020) were significantly enriched in the D14 vs. D28 group. The NF-κB pathway mainly modulates the balance of intestinal inflammatory response, while the calcium signaling pathway participates in the regulation of epithelial cell physiological activities. Jointly, they maintain the dynamic immune homeostasis of the mature cecum (Figure 10B); primary immunodeficiency (ko05340) and rheumatoid arthritis (ko05323) related pathways were significantly enriched in the D1 vs. D28 group (Figure 10C), confirming that the maturation of immune function and the improvement of the hematopoietic system are the core characteristics of cecal development during the brooding period.

### 3.8. Integration of Metabolomic and Transcriptomic Data and Screening of Core Pathways

**Figure 11:**
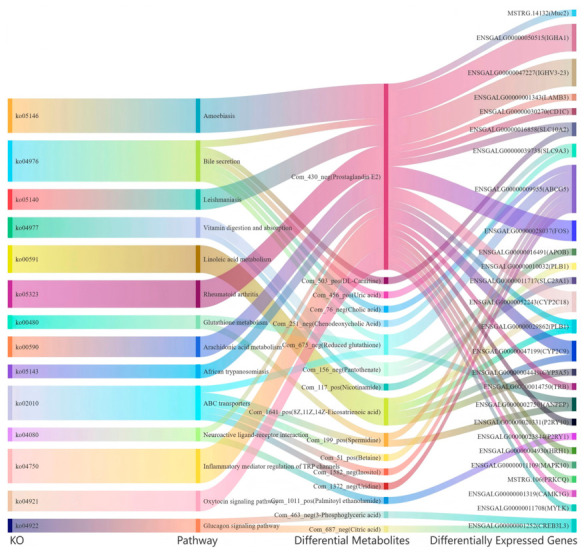
Integrated analysis of KEGG significantly enriched pathways, significantly differential abundance metabolites and significantly differentially expressed genes in cecal tissue of Liangshan Yanying chickens.

To systematically interpret cecal developmental mechanisms at transcriptional and metabolic levels, we integrated KEGG annotation data of differential metabolites and differentially expressed genes for multi-omics correlation analysis. By intersecting 84 core metabolic pathways and 290 core transcriptomic pathways, we obtained 53 shared KEGG pathways, with a data missing rate of 1.82%. These common pathways were classified into five primary categories: organismal systems, metabolism, human diseases, environmental information processing and cellular processes. Among them, metabolic pathways accounted for 52.8% (28 pathways), being the largest category and dominated by carbohydrate metabolism, amino acid metabolism and lipid metabolism; organismal systems pathways accounted for 26.4% (14 pathways), being the second largest category with the core of endocrine and digestive system pathways. The first two categories of pathways accounted for a total of 79.2%, serving as the core regulatory directions of cecal development (detailed information is presented in Supplementary Table S4). Further screening using the multi-dimensional weighted scoring system (Section 2.9) yielded 14 core KEGG pathways from the commonly enriched pathways of metabolites and continuously differentially expressed genes during the brooding period. These pathways corresponded to 16 differential metabolites and 26 differential genes (Figure 11). Sankey diagram visualization analysis showed that these pathways were connected layer by layer from material transport and nutrient metabolism to immune regulation, comprehensively reflecting the core biological characteristics of cecal tissues in maintaining material metabolic homeostasis and mediating immune-inflammatory responses.

### 3.9. Elucidation of Cecal Developmental Regulatory Mechanisms by Integrated Metabolomic and Transcriptomic Analysis

**Figure 12:**
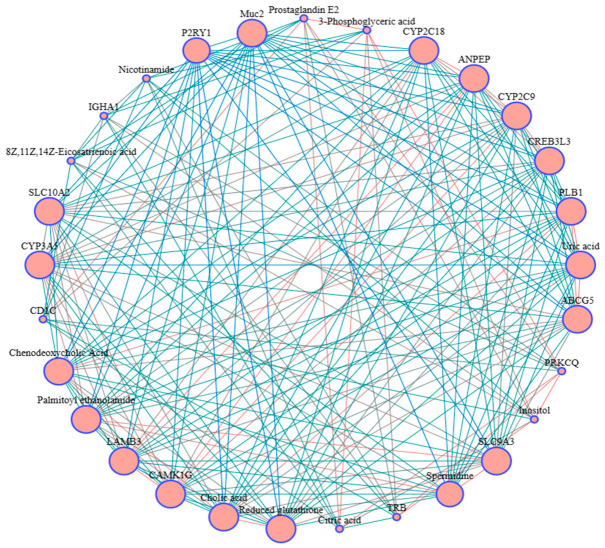
Integrated analysis of key differential metabolites and differentially expressed genes in cecal tissue of Liangshan Yanying chickens during brooding period.

Based on the 14 key KEGG pathways screened by the multi-dimensional weighted scoring system (Section 2.9), a strategy combining hub gene screening and metabolite correlation verification was adopted; molecules with irregular changes in expression level/abundance were excluded, and 12 effective differential metabolites and 19 effective differential genes were identified as the core molecules regulating cecal development. Through a multi-dimensional weighted scoring system, 3 key KEGG pathways, 3 key metabolites and 5 key genes were further screened, on the basis of which an interaction network of key genes-core metabolites-key pathways was constructed (Figure 12).

**Figure 13:**
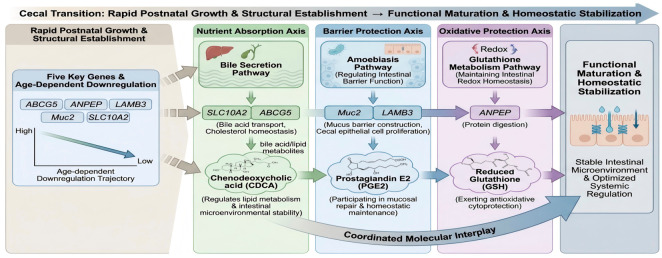
Schematic diagram of molecular mechanisms underlying the regulation of cecal tissue development by key metabolic pathways, metabolites and genes in Liangshan Yanying chickens during brooding period.

The three key pathways were as follows. Bile secretion: This pathway cooperates with hub genes SLC10A2 and ABCG5 to regulate bile acid synthesis, enterohepatic circulation and lipid emulsification. It supports nutrient absorption in early chicks, and maintains intestinal microenvironment stability and lipid metabolism homeostasis in the late brooding stage, providing a material basis for cecal mucosal growth. Glutathione metabolism: This pathway takes reduced glutathione as the core functional metabolite and interacts with ANPEP to eliminate reactive oxygen species in intestinal tissues. It protects cecal epithelial cells from oxidative damage, sustains the integrity of intestinal tissue, and indirectly regulates the activity of immune cells. Amoebiasis: This pathway does not directly participate in pathogen infection. It modulates the expression of barrier-related genes MUC2 and LAMB3, regulates the synthesis of intestinal mucus and the structure of epithelial cell connections, and promotes the morphological development and functional maturation of the cecal mucosal barrier. The three key metabolites were prostaglandin E2 (involved in mucosal repair and homeostasis maintenance), reduced glutathione (exerting antioxidant protection effects), and chenodeoxycholic acid (regulating lipid metabolism and intestinal microenvironment stability). The five key genes were *ABCG5*, *ANPEP*, *LAMB3*, *MUC2* and *SLC10A2*, all showing an age-dependent continuous downregulation trend, which are involved in cecal epithelial cell proliferation, mucus barrier construction, protein digestion, bile acid transport and cholesterol balance regulation, respectively. Their expression patterns are highly consistent with the developmental law of the intestinal tract from rapid developmental construction to functional mature homeostasis. The above key factors jointly form three synergistic regulatory networks: the nutrient absorption axis (Bile secretion→*SLC10A2*/*ABCG5*→bile acid/lipid metabolites), the barrier protection axis (Amoebiasis→*MUC2*/*LAMB3*→Prostaglandin E2) and the oxidative protection axis (Glutathione metabolism→*ANPEP*→Reduced glutathione). The molecular synergistic effects of each regulatory network jointly drive the transformation of the cecum from rapid growth to functional maturation and homeostasis (Figure 13).

### 3.10. qRT-PCR Validation of Key Differentially Expressed Genes

**Figure 14:**
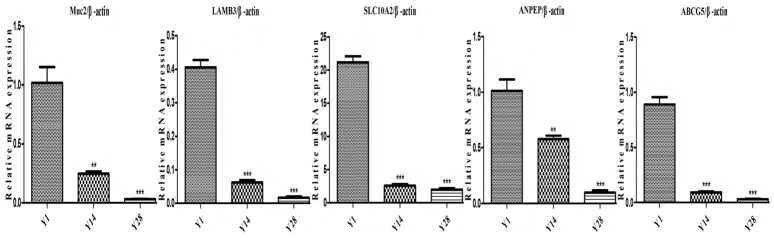
qRT-PCR validation of key differentially expressed genes in cecal tissue of Liangshan Yanying chickens during brooding period. Note: ** indicates significant difference at *p* < 0.01; *** indicates extremely significant difference at *p* < 0.001.

With β-actin as the internal reference gene, the expression levels of the five key genes were validated by qRT-PCR with three technical replicates set for each sample; the relative expression levels of target genes were calculated using the 2^−ΔΔCt^ method. Results showed that the relative expression trends of the five key genes in cecal tissues at the three ages were completely consistent with the change trends of transcriptomic FPKM values without significant differences (Figure 14), confirming the reliability of the transcriptome sequencing data and the accuracy of the DEG screening results in this study, and thus providing a solid molecular basis for subsequent gene function verification.

## 4. Discussion

An integrated analysis of metabolomics and transcriptomics systematically elucidates the characteristics of metabolic remodeling and the underlying molecular regulatory mechanisms in the cecum of Liangshan Yanying chickens during the brooding period (D1–D28). This work preliminarily infers that day 14 is a potential pivotal stage for the maturation of cecal metabolic function and transcriptional regulation. Combined with metabolomic data, we speculate this time point may also be critical for cecal microbiota colonization, while direct microbiome verification is still lacking. The core pathways, metabolites and genes involved in cecal development were further discovered, which establishes an innovative multi-omics framework for investigating intestinal development in native poultry breeds.

The brooding period represents a critical window for the morphological construction and functional improvement of the intestinal tract in poultry. As the core organ of the intestinal microecology in poultry, the developmental status of the cecum directly determines the somatic nutrient absorption capacity and immune defense capacity [17,18].This study finds that the metabolite composition of the cecum in Liangshan Yanying chickens exhibits significant temporal differences with increasing age; the shift from endogenous energy supply to exogenous nutrient metabolism is completed during D1 to D14, which is highly consistent with the physiological processes of feeding initiation, intestinal digestive enzyme system activation and initial microbial colonization in chicks after hatching [19,20]. The upregulation of short-chain fatty acid derivatives and amino acid metabolites further confirms the gradual establishment of nutrient absorption capacity in the intestinal tract of brooding chickens. The significant upregulation of lipid metabolism products during D14 to D28 suggests that the metabolic focus of the cecum shifts to mucosal barrier construction and energy storage; the accumulation of unsaturated fatty acids and phospholipids provides material basis for the synthesis of intestinal epithelial cell membranes, which is consistent with the characteristics of accelerated growth rate and elevated somatic energy demand in the late brooding period of poultry [21,22]. The three types of temporal expression trends of metabolites identified by Mfuzz clustering analysis further reveal the metabolic law of endogenous nutrient consumption-microbial metabolic activation-enhanced lipid synthesis in the cecum during the brooding period. Furthermore, based on the dynamic variations of microbe-associated metabolites, we infer that 14 days of age represents a critical stage for the maturation of cecal microbial colonization in Liangshan Yanying chickens. Notably, this conclusion is drawn from metabolomic data and requires direct validation through microbiome sequencing. The findings also provide an important time reference for optimizing cecal development and feeding management of Liangshan Yanying chickens by modulating intestinal microecology.

Transcriptomic analysis results show that gene expression in cecal tissues exhibits significant temporal remodeling characteristics during the brooding period; the screening of continuously differentially expressed genes provides core clues for elucidating the molecular targets of cecal development. The significant enrichment of the intestinal IgA immune network pathway throughout the brooding period confirms that the establishment of mucosal immune function is a core characteristic of cecal development [23]. The enrichment of hematopoietic cell lineage and cytokine-related pathways during D1 to D14 suggests that this stage is a critical period for immune cell proliferation and immune signal network construction. The significant enrichment of the NF-κB signaling pathway during D14 to D28 indicates that the late stage of cecal development enters a period of fine regulation of immune homeostasis, which is consistent with the developmental law of immune function from establishment to maturation in the brooding period of poultry [24,25,26]. The five key genes (*ABCG5*, *ANPEP*, *LAMB3*, *MUC2* and *SLC10A2*) screened in this study all show an age-dependent continuous downregulation trend, whose functions cover intestinal structural construction [27,28,29,30,31], barrier protection [32,33,34], nutrient digestion [32,35] and material transport [36,37], respectively. This expression pattern reflects the natural developmental law that the expression levels of core genes highly expressed in the early stage of the cecum fall back to a steady state after the completion of morphological and functional construction, which has a certain conservation with the molecular regulatory characteristics of intestinal development in mammals, and also provides candidate genes for the excavation of molecular markers related to excellent traits of local poultry breeds. In addition, the transcriptome analysis adopted 3 biological replicates per group, which is a common setting in current livestock and poultry transcriptome studies. Relative to the 10 replicates for metabolomics, fewer transcriptome replicates may slightly decrease the statistical power to screen genes with mild expression changes. Subsequent studies can appropriately increase the number of biological replicates to improve the detection sensitivity of transcriptome data.

Integrated analysis of metabolomics and transcriptomics is a core means to elucidate the molecular mechanisms of complex biological processes [38,39,40,41]. The three synergistic regulatory networks constructed in this study realize the accurate correlation between genes and metabolites during cecal development in Liangshan Yanying chickens for the first time. The layer-by-layer connection of the nutrient absorption axis, barrier protection axis and oxidative protection axis confirms that cecal development is a complex biological event regulated synergistically by multiple processes such as nutrient metabolism, structural construction and immune defense. Among them, the core roles of the Bile secretion pathway and Glutathione metabolism pathway reflect that nutrient metabolic homeostasis and redox homeostasis are the basic guarantees for cecal development; the Amoebiasis pathway indirectly participates in the morphological development of the cecum by regulating intestinal barrier-related genes, revealing the potential role of immune-related pathways in intestinal structural construction. The interaction between key metabolites (Prostaglandin E2, Reduced glutathione) and key genes (*LAMB3*, *MUC2*) further clarifies the regulatory logic of gene regulation of metabolite synthesis and metabolite-mediated realization of physiological functions, thus providing specific action targets for optimizing cecal development through exogenous regulation of the expression level/abundance of key molecules in subsequent research.

This study has certain limitations: Sampling and microbial detection limitation: Only three discrete time points (1, 14 and 28 days of age) were set in this study, and no dense time-point sampling was performed around day 14. In addition, the inference of microbial colonization characteristics was completely based on metabolomic data, and no intestinal microbiome sequencing was carried out for direct verification. Data validation limitation: qRT-PCR only verified the reliability of transcriptomic data. The metabolomic results and the predicted gene-metabolite-pathway regulatory networks were obtained by correlation analysis, without independent in vitro cell experiments, metabolite intervention or gene manipulation verification. Sample size limitation: Transcriptome analysis adopted 3 biological replicates per group, which is less than the 10 replicates for metabolomics. A small number of replicates may reduce the detection ability of weakly differentially expressed genes and slightly affect statistical power. Omics coverage limitation: This study only combined transcriptomics and metabolomics. The integration of proteomics is needed in the future to construct a more comprehensive regulatory network. Meanwhile, the biological functions of candidate molecules still need to be verified by cellular and animal models. In summary, the characteristics of metabolic remodeling and the underlying molecular regulatory mechanisms in the cecum of Liangshan Yanying chickens during the brooding period revealed in this study not only improve the molecular theory of early intestinal development in poultry, but also provide practical guidance for the optimization of feeding and management of Liangshan Yanying chickens during the brooding period: nutritional regulation can be adopted before 14 days of age to promote intestinal microbial colonization and the establishment of digestive and absorption functions; exogenous addition of antioxidants and lipid nutrients can be applied after 14 days of age to facilitate the maturation of the intestinal mucosal barrier and the construction of immune homeostasis. Meanwhile, the screened key genes can be used as molecular markers to carry out disease-resistant breeding research on Liangshan Yanying chickens, so as to improve the breeding efficiency and stress resistance of this local poultry breed.

In this study, qRT-PCR was used to verify the expression of key differentially expressed genes, which confirmed the reliability of transcriptomic data. However, no independent in vitro or in vivo experiments were conducted to verify the metabolomic results and the proposed gene-metabolite-pathway regulatory networks. The interaction relationships among key genes, core metabolites and signaling pathways are only predicted based on multi-omics correlation analysis. In follow-up research, cell models, gene interference/overexpression experiments and metabolite intervention tests will be carried out to further validate the authenticity of the regulatory networks.

## 5. Conclusions

This study adopted an integrated widely targeted metabolomic and transcriptomic strategy to explore cecal metabolic remodeling and molecular regulatory mechanisms in Liangshan Yanying chickens during the 1–28-day brooding period. Significant age-dependent changes were identified in cecal metabolites and gene expression, and 14 days post-hatch was confirmed as a key node for cecal metabolic maturation, transcriptional regulation and intestinal microbial colonization. Cecal metabolism exhibited obvious stage-specific characteristics: chickens transformed from endogenous energy metabolism to exogenous nutrient metabolism at 1–14 days of age, while lipid metabolism dominated cecal physiological processes from 14 to 28 days of age. Four core KEGG pathways regulating cecal development were identified, together with one hub metabolite (Prostaglandin E2) and five hub genes (ABCG5 included). Three synergistic regulatory axes modulating nutrient absorption, intestinal barrier and antioxidation were constructed, which drive cecal development from structural growth to functional homeostasis. qRT-PCR results verified the credibility of transcriptomic data.

This work has two limitations: the experiment was conducted at a single altitude without lowland breed or altitude-gradient controls, and the omics-based regulatory networks require further functional validation. Collectively, this study clarifies the molecular mechanism of early cecal development in this indigenous chicken breed. It provides novel insights into poultry intestinal health regulation, and supports brooding management and molecular selective breeding of Liangshan Yanying chickens.

## Data Availability

All raw data generated in this study have been deposited in the Genome Sequence Archive (GSA) at the China National Center for Bioinformation (CNCB) (https://ngdc.cncb.ac.cn/gsa/) (Accessed on 5 July 2025), with the transcriptome data assigned the accession number PRJCA042510. Additional study data have been deposited in the Open Archive for Miscellaneous Data (OMIX) at CNCB/Beijing Institute of Genomics, Chinese Academy of Sciences (https://ngdc.cncb.ac.cn/omix) (Accessed on 2 February 2026), under the accession number OMIX015283.

## Supplementary Materials

The following supporting information can be downloaded at: https://www.mdpi.com/article/10.3390/vetsci13060594/s1, Table S1: Optimal Nutrient Levels of Pre-starter and Starter Diets for Broiler Chicks During the Starter Phase; Table S2: List of Q-PCR verification primer sequences for some genes; Table S3: Sequencing data quality and gene sequencing rate of cecal tissue samples from Liangshan Yanying Chickens at different ages; Table S4: Statistics of jointly enriched pathways by differential metabolites and differentially expressed genes in cecal tissue of Liangshan Yanying chickens at 1, 14, and 28 days of age.
