# Supplementary material for: Multi-Omics Analysis Reveals Age-Dependent Metabolic Remodeling and Immune Maturation in the Cecum of Liangshan Yanying Chickens
Source: Vet Sci. 2026 Jun 18;13(6):594. doi: 10.3390/vetsci13060594 (PMC13308322; doi:10.3390/vetsci13060594)
Supplement: Supplementary file 1 [file vetsci-13-00594-s001.zip › Supplementary Table S1.pdf]

Supplementary Table 1 Optimal Nutrient Levels of Pre-starter and Starter Diets for Broiler Chicks During the Starter Phase

Nutrient Indicators	Units	Pre-starter Diet (1-14 days of age)	Starter Diet (15-28 days of age)	Standard Basis and Adjustment Notes
Metabolizable Energy (ME)	MJ/kg (kcal/kg)	12.54 (3000)	12.75 (3050)	NY/T 33-2004 Table 4 (baseline value for 0-3 weeks of age); Energy appropriately increased for 15-28 days of age to match the increased feed intake and growth rate of chicks
Crude Protein (CP)	%	21.5	21.0	Adjusted based on the lower limit (21.0%) in NY/T 33-2004 Table 4; Increased by 0.5 percentage points for 1-14 days of age to adapt to the physiological characteristics of immature intestinal tract and low digestive enzyme activity in chicks, ensuring protein supply efficiency
Protein-Energy Ratio (CP/ME)	g/MJ	17.14	16.47	Calculated based on crude protein and metabolizable energy values, meeting the core requirement of energy-nitrogen balance for broiler chicks during the starter phase to avoid protein waste or energy deficiency
Calcium (Ca)	%	1.00	0.95	Fixed value in NY/T 33-2004 Table 4; Slightly decreased for 15-28 days of age to match the skeletal development rhythm of chicks and reduce calcium deposition burden
Total Phosphorus (TP)	%	0.65	0.63	Fixed value in NY/T 33-2004 Table 4; Fine-tuned for 15-28 days of age to form a balanced ratio with available phosphorus
Available Phosphorus (AP)	%	0.45	0.43	Core value in NY/T 33-2004 Table 4; Maintained at a high level due to insufficient phytase secretion in chicks to ensure phosphorus absorption efficiency
Lysine (Lys)	%	1.15	1.10	Based on NY/T 33-2004 Table 4 (1.10%); Increased by 0.05 percentage points for 1-14 days of age. As the first limiting amino acid, it meets the rapid growth needs of chicks
Lysine-Energy Ratio (Lys/ME)	g/MJ	0.917	0.863	Calculated value, conforming to the ratio law of amino acids and energy for broiler chicks during the starter phase

Methionine (Met)	%	0.52	0.50	Fixed value in NY/T 33-2004 Table 4; Slightly increased for 1-14 days of age to ensure the supply of sulfur-containing amino acids and promote intestinal mucosal development
Methionine + Cystine (Met+Cys)	%	0.92	0.90	Fixed value in NY/T 33-2004 Table 4; Slightly increased for 1-14 days of age to meet the developmental needs of immune organs in chicks
Threonine (Thr)	%	0.77	0.75	Fixed value in NY/T 33-2004 Table 4; Slightly increased for 1-14 days of age. As a key amino acid for intestinal mucosal repair, it alleviates intestinal stress in chicks
Tryptophan (Trp)	%	0.23	0.22	Fixed value in NY/T 33-2004 Table 4; Slightly increased for 1-14 days of age to regulate feed intake and neural development of chicks, improving feed intake
