# Supplementary material for: Multi-Omics Analysis Reveals Age-Dependent Metabolic Remodeling and Immune Maturation in the Cecum of Liangshan Yanying Chickens
Source: Vet Sci. 2026 Jun 18;13(6):594. doi: 10.3390/vetsci13060594 (PMC13308322; doi:10.3390/vetsci13060594)
Supplement: Supplementary file 1 [file vetsci-13-00594-s001.zip › Supplementary Table S2.pdf]

Supplementary Table S2 List of Q-PCR verification primer sequences for some genes

Gene Name	Sequence（5’-3’）	Size
chicken β-actin	GTGTGATGGTTGGTATGGGC<br>CTCTGTTGGCTTTGGGGTTC	225bp
chicken ABCG5	AAGGTTGATGCGGTTATGGC<br>CGAGACGATCTGGTTTGCAG	201bp
chicken ANPEP	TGGTTGAATGAGGGCTTTGC<br>TGCTGGGGTGTTGATCTCAT	177bp
chicken SLC10A2	CATTACTGCTGTGGTTGGGG<br>ACGACATCTGCTCCAAGACA	151bp
chickenMUC2	AATGCACCTGGACAGACTGG<br>ACTGGTGTGCTGATTGTCGT	343bp
chickenLAMB3	CCAGCAGCTTCTATGCAGTG<br>ATTGTAGAGGGCAGCACAGT	173bp
