# Supplementary material for: Multi-Omics Analysis Reveals Age-Dependent Metabolic Remodeling and Immune Maturation in the Cecum of Liangshan Yanying Chickens
Source: Vet Sci. 2026 Jun 18;13(6):594. doi: 10.3390/vetsci13060594 (PMC13308322; doi:10.3390/vetsci13060594)
Supplement: Supplementary file 1 [file vetsci-13-00594-s001.zip › Supplementary Table S3.pdf]

Supplementary Table S3: Sequencing data quality and gene sequencing rate of cecal tissue samples from Liangshan Yanying Chickens at different ages

Sample	Raw Data Yield (bp)	BF_Q20（%）	BF_Q30（%）	BF_N（%）	BF_GC（%）	Filtered Data Yield (bp)	AF_Q20（%）	AF_Q30（%）	AF_N（%）	AF_GC（%）	Number of Reference Genes	Reference Gene Sequencing Rate (%))	Number of Novel Genes	Novel Gene Sequencing Rate (%))	Total Number of Genes	Total Gene Sequencing Rate (%)
YMZ1-1	6,426,149,400	6,250,400,860 (97.27%)	5,968,550,825 (92.88%)	68,719 (0.00%)	3,041,246,490 (47.33%)	6,348,158,837	6,188,695,477 (97.49%)	5,914,074,894 (93.16%)	68,057 (0.00%)	2,999,551,675 (47.25%)	16,878	14,407 (85.36%)	1,087	950 (87.40%)	17,965	15,357 (85.48%)
YMZ1-2	6,653,836,500	6,435,679,089 (96.72%)	6,105,713,101 (91.76%)	69,528 (0.00%)	3,140,577,513 (47.20%)	6,580,923,993	6,382,271,881 (96.98%)	6,059,928,086 (92.08%)	68,813 (0.00%)	3,100,426,364 (47.11%)	16,878	14,471 (85.74%)	1,087	947 (87.12%)	17,965	15,418 (85.82%)
YMZ1-3	7,777,319,100	7,593,465,952 (97.64%)	7,274,640,003 (93.54%)	85,874 (0.00%)	3,652,299,433 (46.96%)	7,694,069,932	7,527,640,796 (97.84%)	7,216,339,179 (93.79%)	85,041 (0.00%)	3,606,298,612 (46.87%)	16,878	14,602 (86.51%)	1,087	976 (89.79%)	17,965	15,578 (86.71%)
YMZ14-1	6,121,803,000	5,952,379,312 (97.23%)	5,681,236,973 (92.80%)	65,640 (0.00%)	2,942,895,971 (48.07%)	6,059,982,048	5,904,878,965 (97.44%)	5,639,800,063 (93.07%)	64,965 (0.00%)	2,908,552,288 (48.00%)	16,878	14,503 (85.93%)	1,087	951 (87.49%)	17,965	15,454 (86.02%)
YMZ14-2	6,010,971,300	5,815,313,246 (96.74%)	5,520,058,039 (91.83%)	64,304 (0.00%)	2,887,865,207 (48.04%)	5,945,182,564	5,766,232,149 (96.99%)	5,477,870,121 (92.14%)	63,765 (0.00%)	2,851,811,391 (47.97%)	16,878	14,497 (85.89%)	1,087	960 (88.32%)	17,965	15,457 (86.04%)
YMZ14-3	6,776,961,900	6,571,012,519 (96.96%)	6,253,214,298 (92.27%)	71,726 (0.00%)	3,229,756,979 (47.66%)	6,701,440,838	6,513,416,800 (97.19%)	6,203,177,876 (92.56%)	71,066 (0.00%)	3,188,301,593 (47.58%)	16,878	14,564 (86.29%)	1,087	978 (89.97%)	17,965	15,542 (86.51%)
YMZ28-1	5,831,885,100	5,644,069,395 (96.78%)	5,362,208,212 (91.95%)	60,998 (0.00%)	2,800,581,524 (48.02%)	5,760,699,965	5,589,601,979 (97.03%)	5,314,902,832 (92.26%)	60,247 (0.00%)	2,761,157,155 (47.93%)	16,878	14,569 (86.32%)	1,087	967 (88.96%)	17,965	15,536 (86.48%)
YMZ28-2	6,911,534,100	6,705,818,648 (97.02%)	6,387,233,711 (92.41%)	72,702 (0.00%)	3,317,298,449 (48.00%)	6,837,185,727	6,648,944,109 (97.25%)	6,337,721,931 (92.69%)	71,993 (0.00%)	3,276,058,205 (47.92%)	16,878	14,721 (87.22%)	1,087	977 (89.88%)	17,965	15,698 (87.38%)
YMZ28-3	6,498,226,500	6,301,257,428 (96.97%)	5,999,705,590 (92.33%)	69,713 (0.00%)	3,109,603,647 (47.85%)	6,419,829,512	6,240,532,776 (97.21%)	5,946,598,286 (92.63%)	68,913 (0.00%)	3,066,198,065 (47.76%)	16,878	14,663 (86.88%)	1,087	973 (89.51%)	17,965	15,636 (87.04%)
