# Supplementary material for: Multi-Omics Analysis Reveals Age-Dependent Metabolic Remodeling and Immune Maturation in the Cecum of Liangshan Yanying Chickens
Source: Vet Sci. 2026 Jun 18;13(6):594. doi: 10.3390/vetsci13060594 (PMC13308322; doi:10.3390/vetsci13060594)
Supplement: Supplementary file 1 [file vetsci-13-00594-s001.zip › Supplementary Table S4.pdf]

Supplementary Table S4. Statistics of jointly enriched pathways by differential metabolites and differentially expressed genes in cecal tissue of Liangshan Yanying chickens at 1, 14, and 28 days of age

KO	co-Pathway	Pvalue	Qvalue	KO	co-Pathway	Pvalue	Qvalue
ko05146	Amoebiasis	0.3336	0.2131	ko00052	Galactose metabolism	1.8315	0.6337
ko04976	Bile secretion	0.5830	0.2842	ko01230	Biosynthesis of amino acids	2.4480	0.6412
ko05140	Leishmaniasis	0.3322	0.2874	ko05230	Central carbon metabolism in cancer	1.6253	0.6632
ko04977	Vitamin digestion and absorption	1.1244	0.3157	ko00250	Alanine, aspartate and glutamate metabolism	1.7369	0.6800
ko04974	Protein digestion and absorption	1.3530	0.3200	ko00410	beta-Alanine metabolism	1.5078	0.6856
ko00591	Linoleic acid metabolism	0.9825	0.3228	ko00630	Glyoxylate and dicarboxylate metabolism	1.6147	0.6858
ko05323	Rheumatoid arthritis	0.9085	0.3256	ko04742	Taste transduction	2.7972	0.6868
ko04978	Mineral absorption	1.5624	0.3493	ko01100	Metabolic pathways	2.1128	0.6916
ko00480	Glutathione metabolism	0.6334	0.4154	ko04726	Serotonergic synapse	2.6964	0.6981
ko04964	Proximal tubule bicarbonate reclamation	1.2306	0.4171	ko00561	Glycerolipid metabolism	2.0729	0.7164
ko00590	Arachidonic acid metabolism	1.6797	0.4206	ko00230	Purine metabolism	2.9512	0.7211
ko05143	African trypanosomiasis	1.2900	0.4602	ko00380	Tryptophan metabolism	3.3099	0.7267
ko02010	ABC transporters	0.2873	0.4620	ko00240	Pyrimidine metabolism	3.1393	0.7357
ko04080	Neuroactive ligand-receptor interaction	1.8538	0.4975	ko04924	Renin secretion	2.8139	0.7429
ko00120	Primary bile acid biosynthesis	0.9996	0.5121	ko00270	Cysteine and methionine metabolism	3.5862	0.7487
ko04750	Inflammatory mediator regulation of TRP channels	1.3602	0.5409	ko00051	Fructose and mannose metabolism	2.6414	0.7619
ko00260	Glycine, serine and threonine metabolism	1.7733	0.5450	ko04024	cAMP signaling pathway	3.4819	0.7675
ko04921	Oxytocin signaling pathway	1.4135	0.5478	ko04212	Longevity regulating pathway - worm	2.4105	0.7731
ko00030	Pentose phosphate pathway	1.6943	0.5552	ko04142	Lysosome	1.8777	0.7736
ko04922	Glucagon signaling pathway	1.3551	0.6006	ko04070	Phosphatidylinositol signaling system	2.6765	0.7762
ko01040	Biosynthesis of unsaturated fatty acids	1.7698	0.6077	ko00730	Thiamine metabolism	2.7372	0.7781
ko00760	Nicotinate and nicotinamide metabolism	1.0692	0.6094	ko04923	Regulation of lipolysis in adipocytes	2.7720	0.7964
ko04918	Thyroid hormone synthesis	1.9303	0.6114	ko00430	Taurine and hypotaurine metabolism	2.7380	0.8047
ko01200	Carbon metabolism	2.0102	0.6160	ko05200	Pathways in cancer	2.4873	0.8154
ko00330	Arginine and proline metabolism	1.3629	0.6194	ko00562	Inositol phosphate metabolism	3.1360	0.8441
ko00520	Amino sugar and nucleotide sugar metabolism	1.5209	0.6216	ko00620	Pyruvate metabolism	3.7486	0.8861
ko00340	Histidine metabolism	1.8670	0.6300				
